# Advantages and Disadvantages of the Ketogenic Diet: A Review Article

**DOI:** 10.7759/cureus.9639

**Published:** 2020-08-10

**Authors:** Jennifer T Batch, Sanjay P Lamsal, Michelle Adkins, Senan Sultan, Monica N Ramirez

**Affiliations:** 1 Internal Medicine, Orange Park Medical Center, Orange Park, USA; 2 Radiology, University of Florida Health Jacksonville, Jacksonville, USA; 3 Pharmacy, University of Florida, Gainesville, USA; 4 Endocrinology, Orange Park Medical Center, Orange Park, USA; 5 Pharmacy, University of Florida Health Jacksonville, Jacksonville, USA

**Keywords:** ketogenic diet, low carbohydrate diet, low fat diet, polycystic ovarian syndrome, obesity, diabetes mellitus, insulin resistance

## Abstract

The ketogenic diet (KD) has gained immense popularity during the last decade, primarily because of its successful short-term effect on weight loss. In the United States, KD is utilized in a variety of patient populations for weight management, despite limited evidence regarding its efficacy and risks. This literature review provides an evaluation of data on the benefits and risks associated with the chronic use of KD, including its metabolic, endocrinological, and cardiovascular effects.

## Introduction and background

Obesity is classified based on the body mass index (BMI) of the individual. A BMI of 18.5-24.9 kg/m^2^ is considered to be the normal range, while a BMI of 25.0-29.9 is considered overweight and a BMI ≥30 is classified as obese (further classified as obesity class I if BMI is between 30.0-34.9, class II if BMI is between 35.0-39.9, and class III if BMI is ≥40.0). In 2016, the World Health Organization (WHO) reported that more than 1.9 billion (39%) adults were overweight globally and of these, over 650 million (13%) were obese [1]. Obesity is associated with multiple comorbidities including type 2 diabetes, hypertension, cardiovascular disease (CVD), cancer, sleep apnea, and obesity-hypoventilation syndrome (OHS). The effectiveness of different types of diets based on different macronutrient restrictions has been a topic of debate for the past few years. Some researchers support restriction in carbohydrate (CHO), while others endorse cutting down protein or fats [2].

This review article will focus on the ketogenic diet KD, which is defined as a low-carbohydrate diet (LCD) with a moderate amount of protein restriction to induce ketosis without restricting fat intake [3]. The concept of KD was initially developed in 1921 by Dr. Russel Wilder for the management of refractory seizures in pediatric patients [4]. Originally, the diet consisted of a 4:1 ratio of fat-to-CHO and protein. Fat provides upwards of 90% the caloric intake [5]. All variations of this diet, whether involving animal- or plant-based derivatives, are based on severely restricting overall intake of CHO with a goal of bringing it down to less than 50 g/day. A well-formulated KD limits protein intake moderately to less than 1 g/lb body weight, or 1.5 g/lb body weight for individuals performing heavy exercises. Additionally, the diet does not restrict fat intake while decreasing appetite and caloric intake, resulting in weight loss observed after the initiation of the diet [6].

Following CHO deprivation and depletion of glycogen stores, the body undergoes metabolic changes to provide an energy source for the body through gluconeogenesis and ketogenesis. Gluconeogenesis can be sustained for three days with adherence to an LCD, and subsequently, additional energy sources are necessary to meet the metabolic requirements of the body and brain. This is where the process of ketogenesis becomes indispensable, and the formation of ketone bodies is then used as the primary energy source by cells with mitochondria and, most importantly, the brain [6].

KD has been shown to effectively lead to weight loss, reduction in hyperinsulinemia, and improvement in insulin sensitivity. However, patients diagnosed with diabetes on insulin or oral hypoglycemic agents may suffer severe hypoglycemia if their medication regimen is not properly managed during the initiation of KD. Furthermore, the diet is limited and/or contraindicated in patients with liver failure, pancreatitis, inborn disorders of fat metabolism, primary carnitine deficiency, carnitine palmitoyltransferase deficiency, carnitine translocase deficiency, porphyria, and pyruvate kinase deficiency [6]. Common short-term side effects resulting from the initiation of KD have been referred to as “keto flu,” which encompasses symptoms including fatigue, headache, dizziness, nausea, vomiting, constipation, and low exercise tolerance [6]. Symptoms typically resolve after a few days to weeks as the body adjusts to the low CHO, ketogenic state. Long-term side effects include hepatic steatosis, kidney stones, hypoproteinemia, and vitamin deficiency. While the benefits of following KD have been extensively reported, long-term compliance with KD is a limiting factor. The sustainability of the diet has been called into question, and the prognosis of the diet’s effects after discontinuation must be examined.

## Review

1. Ketogenic diet and cardiovascular risk factors

Dyslipidemia

In a systematic review and meta-analysis of clinical trials performed by Santos et al., a total of 23 randomized controlled trials corresponding to 17 clinical investigations were analyzed. The authors concluded that LCD has positive effects on body weight, BMI, abdominal circumference, blood pressure, high-density lipoprotein cholesterol (HDL-C), triglycerides, glycemia, hemoglobin A1c (HbA1c), insulin, and C-reactive protein (CRP) [2]. However, despite the positive impact on cardiovascular risk factors, there is insufficient data to support KD in the long term as the studies were of relatively shorter duration, ranging from three to 36 months only.

In another meta-analysis performed by Bueno et al., a total of 13 randomized controlled trials were examined. The authors reported statistically significant results in the first six months of intervention, but at longer periods of 12-24 months, the statistical significance of outcomes decreased. In a majority of the studies analyzed, CHO intake was higher than the protocol allowed, which was less than 50 g of CHO per day. These similarities found during the follow-up period were likely a contributing factor to the observed decrease in statistical significance [7]. Also, participants following a very-low-carbohydrate ketogenic diet (VLCKD) had a significantly greater increase in low-density lipoprotein cholesterol (LDL-C) levels when compared to participants following a low-fat diet (LFD) (95% CI: 0.04 to 0.2; p=0.002). This increase in LDL-C may subsequently lead to the development of accelerated atherosclerosis and increases the risks associated with CVD.

The lack of evidence regarding long-term cardiovascular implications indicates that making recommendations against or in favor of KD should be a topic of further discussion. In a randomized controlled trial performed at the outpatient care of the Philadelphia Veterans Affairs Medical Center among adults of ≥18 years old with a BMI of ≥35 kg/m^2^, 64 participants were assigned to an LCD and 68 participants were assigned to a conventional diet. Analysis at one year of initiating the study found favorable metabolic effects on atherogenic dyslipidemia and glycemic control with participants on an LCD compared to participants on a conventional diet [8]. The mean weight change for participants assigned to the LCD group was -5.1 ±8.7 kg, while it was -3.1 ±8.4 kg for the conventional diet group. However, the differences in weight change were not significant [-1.9 kg (95% CI: -4.9 to 1.0 kg), p=0.20]. This reinforces the low sustainability of a long-term LCD and the high likelihood of regaining the weight lost. 

Interestingly, multiple studies mention a “favorable” lipid profile associated with KD because it increases HDL-C and decreases triglyceride levels. Distinguishably, increases are observed with LDL-C and total cholesterol. A review article analyzing five randomized controlled trials concluded that, after six months, individuals assigned to an LCD lost more weight than individuals assigned to an LFD with a weight difference of -3.3 kg (95% CI: -5.3 to -1.4 kg). Nevertheless, this significant difference was not identified after one year of intervention (95% CI: -3.5 to 1.5 kg). Triglyceride levels had a significant decrease [-22.1 mg/dl (95% CI: -38.1 to -5.3 mg/dl)], and HDL-C levels underwent a significant increase [4.6 mg/dl (95% CI: 1.5 to -8.1 mg/dl)] in the LCD group when compared to the LFD group. Conversely, LDL-C and total cholesterol changed more favorably in the LFD group after six months of the intervention [9]. The mechanism postulated for this is mediated through lower CHO intake, inducing suppressed insulin production. Concomitantly, decreased insulin production inhibits 3-hydroxy-3-methyl-glutaryl-coenzyme A (HMG-CoA) reductase activation and stimulates HMG-CoA lyase involved in ketone production. Thus, following a low CHO diet inadvertently leads to increased production of LDL-C and hypothetically promotes atherosclerotic properties.

Differentiation in Fat Sources

As reported by Seidelmann et al. in a prospective cohort study and meta-analysis, it is not only a matter of CHO restriction but also the quality of food ingested. The study’s primary outcome measure was all-cause mortality. After multivariable adjustment and a median follow-up period of 25 years, a U-shape association was observed between the percentage of energy consumed from CHO and mortality [pooled hazard ratio (HR): 1.20 (95% CI: 1.09 to 1.32 for low CHO consumption); pooled HR: 1.23 (95% CI: 1.11 to 1.36 for high CHO consumption)] in the Atherosclerosis Risk in Communities (ARIC) cohort. The authors emphasized that both low (<40%) and high CHO consumption (>70%) conferred higher mortality when compared with moderate CHO intake. Further analysis of the results demonstrated that mortality was worse when fat and protein sources were animal-derived instead of plant-derived. A relationship may exist between decreasing mortality rates and long-term approach considerations in the replacement of CHO with plant-based fats and proteins such as vegetables, nuts, and whole grains [10].

Hypertension

A meta-analysis that included 13 studies aimed to examine whether participants assigned to a VLCKD (diet restriction to ≤50 g CHO/day) would achieve better long-term body weight and cardiovascular risk factor management when compared with participants assigned to a conventional LFD (diet restriction to <30% energy from fat). In the overall analysis, those assigned to the VLCKD group showed a statistically significant decrease in their primary outcome: body weight: -0.91 kg (95% CI: -2.49 to -0.37). Additionally, of the 13 studies, 11 were included in the systolic blood pressure (SBP) and diastolic blood pressure (DBP) subgroup analyses. A statistically greater reduction in DBP [-1.43 mmHg (95% CI: -2.49 to -0.37)] was reported in participants assigned to a VLCKD compared to participants assigned to an LFD. Furthermore, an increase was reported in secondary endpoints: HDL-C: 0.09 mmol/l (95% CI: 0.06 to 0.12), LDL-C: 0.12 mmol/l (95% CI: 0·04 to 0·2), and triacylglycerol (TAG): -0.18 mmol/l (95% CI: -0.27 to -0.08) [7].

In contrast, a meta-analysis of 11 randomized controlled trials proposed to assess the effects of LCD vs LFD on weight loss and risk factors of CVD reported that cardiovascular risk factors such as SBP and DBP were not found to be statistically significant among both groups. A dietary intervention of six months or longer was implemented, and participants assigned to the LCD group had greater reductions in body weight [-2.17 kg (95% CI: -3.36 to -0.99)] and TAG [-0.26 mmol/l, (95% CI: -0.37 to -0.15)]. On the other hand, increases in LDL-C [0.16 mmol/l (95% CI: 0.003 to 0.33)] and HDL-C [0.14 mmol/l, (95% CI: 0.09 to 0.19)] were observed [11].

2. Effects of the ketogenic diet on endocrinology and metabolism

Polycystic Ovarian Syndrome

KD has been postulated to positively impact women diagnosed with polycystic ovarian syndrome (PCOS). Women with PCOS experience symptoms of irregular/absent menses, infertility, obesity, and other phenotypical effects of hyperandrogenism such as hirsutism. PCOS is closely associated with other metabolic and endocrinological irregularities, which include insulin resistance, hyperinsulinemia, type 2 diabetes mellitus, dyslipidemia, and hyperandrogenism [12]. PCOS is accompanied by key features such as insulin resistance, androgen excess, and abnormal gonadotropin dynamics. In turn, treatment is targeted towards improving insulin resistance, weight loss, decreasing luteinizing hormone (LH) and follicular stimulating hormone (FSH) ratios, and excess androgens. A study by Mavropoulos et al. implemented KD for women between the ages of 18-45 years diagnosed with PCOS, with a BMI greater than 27 kg/m2, and no other serious medical conditions. Participants adhered to a six-month period of strict KD consisting of less than 20 g of CHO per day with unlimited consumption of animal-based foods. After 24 weeks, the results of the study (pre- and post-design) showed a statistically significant decrease in fasting serum insulin (23.5 to 8.2, p=0.002), LH-to-FSH ratio (2.23 to 1.21, p≤0.05), and free testosterone (2.19 to 1.70, p≤0.05). Furthermore, the study subjects had an overall mean body weight change from baseline of -12.1% and a mean decrease in BMI of 4.0 kg/m2 (p=0.0006) [12]. Despite the results of the pilot study demonstrating a positive impact, there are limitations in generalization due to the small sample sizes of the study.

A crossover study by Gower et al. included participants with PCOS who were randomly assigned to either a standard diet or an LCD. The results demonstrated that LCD can lead to decreases in glycemia, fasting insulin, testosterone, and insulin sensitivity. However, the study had limitations due to the broad age range of participants and the small sample size, thereby rendering it inadequate in terms of generalizability [13]. Similar results were reported by Paoli et al., with significant reductions in BMI, glycemia, insulin, LDL-C, HDL-C, triglycerides, LH, testosterone, and dehydroepiandrosterone sulfate (DHEAS). Even though a reversal of the LH-to-FSH ratio was observed initially, it was not reported after 12 weeks. Limitations of this study include small sample size, single-arm design, lack of infertility measurements, and a short intervention time interval [14].

Diabetes Mellitus and Insulin

The term “Ketogenic Diet” may lead to apprehension in diabetic patients given its association with the well-known, life-threatening condition of ketoacidosis. It is important to note that during nutritional ketosis, the concentrations of beta-hydroxybutyrate and acetone are of low levels and do not cause any alterations in the pH of blood [6]. Management of type 1 and type 2 diabetes generally consists of medication adjustments targeted toward glycemic control and an HbA1c level of <7%. As mentioned previously with regard to patients with PCOS, the same benefits of following a VLCKD apply to patients with diabetes as well. They include decreased glycemia, lower levels of fasting insulin, decreased insulin resistance, and potentiating decreased requirements of insulin and/or oral glycemic medications. Thus, VLCKD has become popular among patients suffering from diabetes and obesity; nevertheless, the appropriateness of this diet is still debated.

In an outpatient clinic study by Yancy et al., overweight patients diagnosed with type 2 diabetes were made to follow a VLCKD throughout 16 weeks with the primary outcome measure of monitoring blood glucose control through HbA1c levels. The 28 participants enrolled were restricted to less than 20 g of CHO per day. At the end of the 16-week timeframe, HbA1c decreased from 7.5 ±1.4% to 6.3 ±1.0% (p<0.001). The absolute decrease in HgA1c was approximately 1.0% in 11 participants (52%). The relative decrease in HgA1c from baseline was >10% in 14 participants, and >20% in six participants. Furthermore, seven participants had their baseline diabetic medications discontinued, 10 participants had their baseline medications decreased, while four participants had unchanged requirements regarding baseline medications [15]. 

A similar observational study was performed by Leow et al. to evaluate the glycemic benefits of a VLCKD in patients diagnosed with type 1 diabetes. The study had a total of 11 eligible participants based on the study inclusion criteria, which included type 1 diabetes of ≥2 years, not taking any medications other than insulin, fasting blood beta-hydroxybutyrate levels of ≥0.4 mmol/l, and C-peptide levels of <0.05. Participants were required to follow a VLCKD with ingestion of less than 55 g of CHO a day for more than six months. The median duration of intervention was 1.5 years (range: 0.6-3 years). All participants had their HbA1c, C-peptide, beta-hydroxybutyrate levels, lipoprotein profile, markers of liver and kidney function, height, body mass, and blood pressure measurements taken after overnight fasting. The participants were found to have a mean HbA1c of 5.3% ±0.4%; mean and median blood glucose levels determined from continuous glucose monitoring were 5.8 ±1.2 and 5.5 (3.1-8.4) mmol/l, respectively. Daily blood glucose variability, expressed as standard deviation (SD) and coefficient of variation, was 1.5 ±0.7 mmol/l and 26.4% ±8.0%, respectively. The mean and median magnitude of postprandial blood glucose excursions were 0.8 ±1.5 and 0.5 (0-2.2) mmol/l, respectively. Hypoglycemic events were detected with continuous glucose monitoring; patients experienced 0.9 (0.0-2.0) episodes of hypoglycemia per day, defined as blood glucose levels of <3.0 mmol/l [5]. The results of this study indicate that adherence to KD in type 1 diabetics is associated with well-controlled HbA1c levels and minimal glycemic variability. Although this shows some evidence regarding the normalization of HbA1c, the diet comes with increased risks of hypoglycemic episodes. Hence, it is important to emphasize that insulin regimens, as well as oral hypoglycemic agents, must be closely monitored and adjusted in any diabetic patient following a VLCKD regimen. 

Long-term adherence to KD is a major challenge and that is why this type of diet is considered non-sustainable. A comparison of different meta-analyses, review articles, and interventional studies revealed that no uniformity was established in the reported results. The limitations of most studies are attributed to small sample sizes, short duration of interventions, and high participant dropout rates. Due to the above-mentioned issues, even though some studies show positive results, we cannot consider them applicable to the general population; especially given that patients with diabetes or obesity often have other comorbid conditions such as dyslipidemia and CVD.

Obesity

In a controlled study involving 20 participants receiving a nutritional intervention with VLCKD, a significant improvement in anthropometric and biochemical parameters was observed after eight weeks of therapy. This included a reduction in BMI, LDL-C, triglycerides, insulinemia, and liver transaminases. Additionally, it was reported that VLCKD also reduced inflammation [16]. Limitations once again include small sample size and a short period of intervention. In a meta-analysis of 11 studies, significant weight reductions were reported in the LCD groups when compared to LFD groups. Interestingly, the authors mentioned that this was attributed to lower energy intake rather than the macronutrient composition [11].

The aim of The Diet Intervention Examining the Factors Interacting with Treatment Success (DIETFITS) randomized clinical trial was to determine the effect of healthy low-fat (HLF) diet vs a healthy low-carbohydrate (HLC) diet on weight change and if genotype pattern or insulin secretion were related to the dietary effects on weight loss. The clinical trial, involving 609 overweight adults, did not demonstrate any results of statistical significance regarding its primary outcome measure, which was weight change with an HLF diet or HLC diet over 12 months. Similarly, neither type of diet showed outcomes of statistical significance in genotype pattern interaction or baseline insulin secretion interaction with 12-month weight loss. It can be assumed that it would be difficult to identify which type of diet is better for any individual. Thus, this leads us to conclude that dietary modifications remain key to successful weight loss [17].

Lastly, a multicenter randomized controlled trial by Ebbeling et al. randomly assigned participants who achieved target weight loss to either a low CHO, moderate CHO, or high CHO diet. This 20-week interventional study found that independent of body weight, total energy expenditure was significantly greater in participants assigned to low CHO diets compared to high CHO diets of similar protein content. Moreover, a significant decrease in metabolic hormonal response with both ghrelin and leptin was reported in the participants assigned to the low CHO diet compared with those assigned to the high CHO diet. These results led the authors to conclude that these metabolic effects and the correlation of dietary quality with energy expenditure may be helpful in the treatment of obesity [18].

## Conclusions

Based on our review, within the first 6-12 months of initiating KD, transient decreases in blood pressure, triglycerides, and glycosylated hemoglobin, as well as increases in HDL and weight loss may be observed. However, the aforementioned effects are generally not seen after 12 months of therapy, as the changes reported in the studies we reviewed are not statistically significant. Further research is warranted to evaluate the long-term implications of KD. Despite the diet's favorable effect on HDL-C, the concomitant increases in LDL-C and very-low-density lipoproteins (VLDL) may lead to increased cardiovascular risks. Additionally, the dietary restrictions required to sustain ketosis may actually lead to its low sustainability. Unfortunately, most available studies lack generalizability and validity due to their small sample sizes and short study durations. Due to the limited amount of robust studies and lack of strong evidence evaluating the diet’s potential risks, recommendations supporting VLCKD in patients with no comorbidities, or cardiometabolic and endocrinologic diseases should be made at the provider’s discretion.
